# Identification of a Novel Enterovirus Species in Rhesus Macaque in China

**DOI:** 10.1038/srep28526

**Published:** 2016-06-22

**Authors:** Yuan-yun Ao, Jie-mei Yu, Cui-yuan Zhang, Yun-yun Xin, Li-li Li, Zhao-jun Duan

**Affiliations:** 1 National Institute for Viral Disease Control and Prevention, China CDC, Beijing 100052, China; 2 The First affiliated Hospital of Hunan Normal University, Changsha 410000, Hunan, China

## Abstract

Recent studies of *Enterovirus* (EV) in nonhuman primates (NHPs), which could act as a source of future emerging human viral diseases, have boosted interest in the search for novel EVs. Here, a highly divergent strain of EV, tentatively named SEV-gx, was identified by viral metagenomic analysis from stool samples of rhesus macaques in China. In total, 27 of 280 (9.6%) faecal samples from rhesus macaques were positive for SEV-gx. Its complete genomic sequence is 7,367 nucleotide (nt). Genomic analyses showed that it has a standard genomic organisation for EVs, being more closely related to EV-J strains (approximately 54.0%, 43.0–44.1%, 52.3–55.2%, 61.1–62.7% and 64.0% amino acids identity in polyprotein, P1, P2 and P3 and combined 2C/3CD regions, respectively). It was also shown to have genome characteristics typical of EVs. Phylogenetic analysis of P1, 2C and 3CD aa indicated that SEV-gx can be classified as a distinct cluster in the EVs. All of this evidence demonstrates SEV-gx is a novel species (tentatively named EV-K) in the EV genus, which contributes to our understanding of the genetic diversity and evolution of EVs. Further studies are needed to investigate the potential pathogenicity of SEV-gx in NHPs and humans.

The family Picornaviridae comprises small vertebrate viruses with positive-sense, single-stranded RNA genomes 7–9.7 kb in size^1^. Picornaviruses are ubiquitous and infect many vertebrate species; they also exhibit a high degree of genetic diversity, including at least 50 viral species grouped into 29 genera (International Committee on Taxonomy of Viruses, ICTV)^1^,^2^.

The genus *Enterovirus* (EV) is part of the family Picornaviridae. The EV genome contains a single open reading frame (ORF) encoding a large polyprotein, flanked by a 5′-untranslated region (UTR) and a 3′-UTR. The polyprotein is cleaved by proteases into structural and nonstructural proteins during replication, namely, the three regions P1, P2 and P3. The capsid proteins encoded by the P1 region are commonly called VP4, VP2, VP3 and VP1. Nonstructural proteins encoded by the P2 (2A–2C) and P3 regions (3A–3D) play a role in protein processing and genome replication^1^. EVs are wellknown for their antigenic and genetic diversity, with more than 300 serotypes having been identified; some of these viruses are human pathogens and circulate widely worldwide^1^. According to the ICTV, the EV genus is subdivided into at least 12 species, including human-infecting species such as human rhinoviruses (RV-A to C) and human EVs (EV-A to D), as well as those infecting animals such as cows (bovine EVs; EV-E and F), pigs (porcine EVs; EV-G), monkeys (simian EVs; EV-H and J)^1^ and other possible new species identified in dromedaries^3^.

In contrast to human EVs, which have been extensively studied, simian EVs have not received much attention. Simian EVs were first isolated in primate cell cultures and tissue specimens used in biomedical research in the 1950s and 1960s from Old World monkeys^4^,^5^,^6^,^7^,^8^,^9^,^10^. To date, although a number of simian EV strains have been identified in nonhuman primates (NHPs) with diarrhoea, there is still no strong evidence for its association with disease in NHPs^10^,^11^,^12^. Some simian isolates were found to be most closely related to human EVs (A13, SV19, SV43 and SV46 in EV-A; SA5 in EV-B), while other distinct simian EVs are suggested to be classifiable as novel species: EV-J (EV103, N125, N203 and SV6), EV-H (SV2, SV4 and SV28)^13^,^14^,^15^,^16^,^17^,^18^ and others^3^. Although the relationships between EVs infecting humans and NHPs are still unclear, there have been numerous reports recently of widely diverse EVs identified in NHPs, which could be a source of future emerging human viral diseases^19^,^20^,^21^,^22^. In addition, the timescales for the original divergence of EV types and species are still unknown. Thus, it is important to identify more novel EV-related viruses in NHPs.

Here, we describe the full-length sequence and detailed genomic organisation of a novel picornavirus (SEV-gx), which was identified in faecal samples from rhesus macaques (*Macaca mulatta*) at LongHu Mountain in Guangxi Province, China, by Miseq high-throughput sequencing. Genomic and phylogenetic analyses demonstrated that this virus belongs to a new species in the *EV* genus in the family Picornaviridae.

## Results

### Detection of SEV-gx

The assembled reads generated a single contig of 1,452 base pairs (bp), which has a maximum amino acid (aa) sequence identity of 59.0% (with 99.0% coverage) with the proteins of the P1 and P2 connection encoded by EV Coxsackievirus A20 (accession number: ABM54526.1), as determined by BLASTX analysis.

### Prevalence of SEV-gx and analysis of the complete VP1 region

A total of 27 of 280 (9.6%) faecal samples from *Macaca mulatta* were positive for SEV-gx, as determined by reverse-transcription polymerase chain reaction (RT-PCR). The sequences of the complete VP1 region amplified from these samples were determined. All of the 27 VP1 sequences showed 100% nucleotide (nt) identity.

### Genomic characterisation

**Figure 1:**
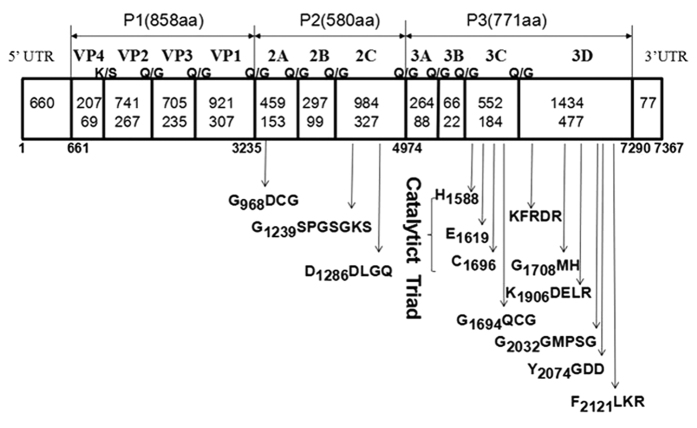
Genomic organisation of SEV-gx with its predicted cleavage sites and conserved *Enterovirus* motifs. The open reading frame is flanked by the 5′-untranslated region (UTR) and the 3′-UTR. Capsid proteins of P1 (VP4, VP2, VP3 and VP1) and nonstructural proteins of P2 (2A, 2B and 2C) and P3 (3A, 3B, 3C and 3D) are shown with nucleotide lengths (upper) and amino acid lengths (lower). The positions of some motifs are shown by the first position in the motif.

Our analysis showed that the assembled complete genome sequence of SEV-gx is 7,367 bp in length, excluding the poly(A) tail. The G + C content is 43.0%, which is within the range of those in the EV genus (from 41.0% to 49.0%). The genome organisation of SEV-gx is similar to those of other EVs. A single ORF of 6,630 nt, encoding a polyprotein of 2,209 aa, was found to be flanked by a 5′-UTR of 660 nt and a 3′-UTR of 77 nt. The full polyprotein includes structural protein P1 of 858 aa (VP4, VP2, VP3 and VP1) and nonstructural proteins P2 of 580 aa (2A, 2B and 2C) and P3 of 771 aa (3A, 3B, 3C^pro^ and 3D^pol^), which shows about 54.0% aa identity with EV-J. The cleavage sites within the polyprotein were predicted to be mainly Gln (Q)/Gly (G) typically processed by 3C^pro^ or 3CD^pro^, except for the cleavage sites at the junction of VP1/VP2 and VP1/2A, which were Lys (K)/Ser (S) and Glu (E)/Gly (G), respectively (Fig. 1), similar to those of picornaviruses.The GenBank accession number for the sequence of SEV-gx is KU587555.

### Genomic analyses

**Figure 2:**
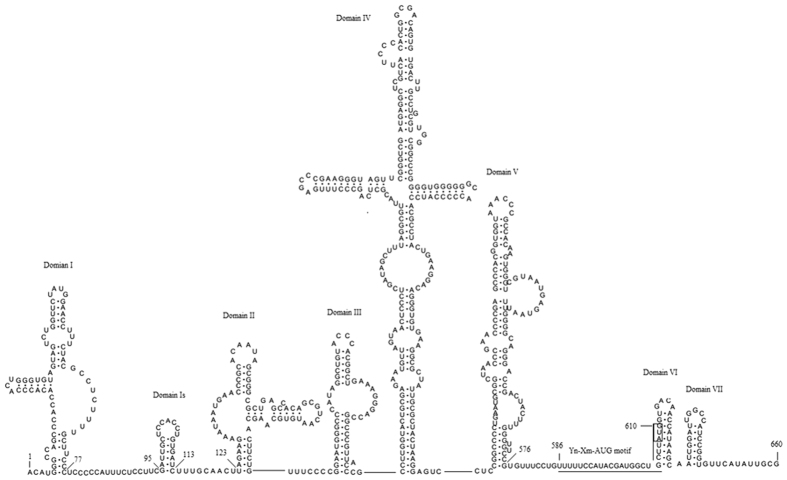
Secondary structure of the predicted internal ribosome entry site (IRES) in SEV-gx. Domain I forms a cloverleaf structure. Domains II, IV, V and VI are the main domains of the IRES. The Yn-Xm-AUG motif is underlined.

The 5′-UTR of SEV-gx shares 38.4–57.1% nucleotide sequence identity with those of strains of other EV species (about 57.1% identity to EV J), but this similarity is much less to those of other picornaviruses (Table 1). The predicted RNA secondary structure of the complete 5′-UTR contained seven domains. Similar to the type I internal ribosome entry site (IRES) of EVs^3^, Domain I (nt 1–77) formed a cloverleaf structure, while five domains, II to VI, were the main domains of IRES element (Fig. 2), which are critical for the initiation of translation in a cap-independent manner^23^. Between domains I and II, there is an additional stem-loop (domain Is), which is also observed in bovine and dromedary EVs^3^. The putative translation initiation site (AUG) of SEV-gx was predicted at the site of nt 661, which was contained in the optimal Kozak context^24^, A_658_AUAUGG. Upstream of the AUG start codon, there was a Yn-Xm-AUG motif (nt 586–610), similar to EVs with type I IRES whose Yn-Xm-AUG motif 25 is located about 30–150-nt upstream of the AUG initiation codon. Based on these findings, SEV-gx IRES can be classified as a type I IRES. There was also no L protein in the polyprotein of SEV-gx, which is similar to previous findings in EVs. The 3′-UTR has no significant similarity to those of other picornaviruses, except 40.2–46.0% nt identity to the strains of EV-C (Table 1) that is within the range of identity between species in EVs (<62.0%)^14^.

The P1 aa sequence of SEV-gx shares 32.2–44.3% nucleotide sequence identity with those of other EV genus strains (the best match being to species EV-J), but this similarity is much less to other picornaviruses (Table 1). A GxxxS/T (G_1_ASVS) myristoylation site was found in the N-terminus of VP0. VP1 of SEV-gx showed the most divergence and included many insertions and deletions (data not shown) in comparison with other picornaviruses, being no more than 32.3% identical at the aa level to those of other EV strains (Table 1). Despite the poor similarity to other strains, the VP1 of SEV-gx also possessed the conserved motifs of PAL(QT)A(AV)ETG and M(FIY)VPPG (P_589_GLNAQETG and M_707_YVPPG motif) found in EV.

The P2 aa sequence of SEV-gx exhibited 52.3–55.2% identity to those of other EV species (Table 1). Although the 2A protein of picornaviruses is a highly variable region (25.3–52.9% aa identity to EVs), a putative catalytic triad of His-Asp-Cys (H_908_D_937_C_969_) identified in EVs was also found^26^. In addition, the conserved GXCG motif (G_968_DCG) was found and formed part of the active site of the protease, suggesting that the 2A protein may function as a viral protease. Like those of other EVs, there were no Asn-Pro-Gly-Pro (NPGP) motifs in 2A and 2B, which are required for co-translational cleavage in avihepatoviruses and avisiviruses^27^. The cysteine-rich region (CX_2_CX_8_CX_4_C) was identified in the SEV-gx 2C protein, identical to the PV1 2C motif, which is used to bind zinc and plays an important role in RNA replication^28^. Similar to all of the other picornaviruses, the NTP binding motif GXXGXGKS (G_1239_SPGSGKS) and the helicase activity motif DDLXQ (D_1286_DLGQ) were also found in the 2C protein^29^.

The identity of the P3 aa sequence of SEV-gx to those of other EV species was 61.1–62.7%, while it showed less than 42.4% aa identity to other picornaviruses. A putative catalytic triad of His-Glu-Cys (H_1588_-E_1619_-C_1696_) was seen in the predicted 3C^pro^ (protease), similar to those in EVs, which differed from those (His-Asp-Cys) in some other genera of picornaviruses. The conserved GXCG (G_1694_QCG) motif was also found in 3C^pro^ of SEV-gx, which formed part of the active site of the viral protease. Similar to those in the EV genus, the putative RNA-binding domain (K_1630_FRDI)^30^ and the motifs of RNA-dependent RNA polymerases (K_1906_DELR, G_2032_GMPSG, Y_2074_GDD and F_2121_LKR)^31^ were also identified in the 3D^pol^ of SEV-gx.

### Phylogenetic analysis

**Figure 3:**
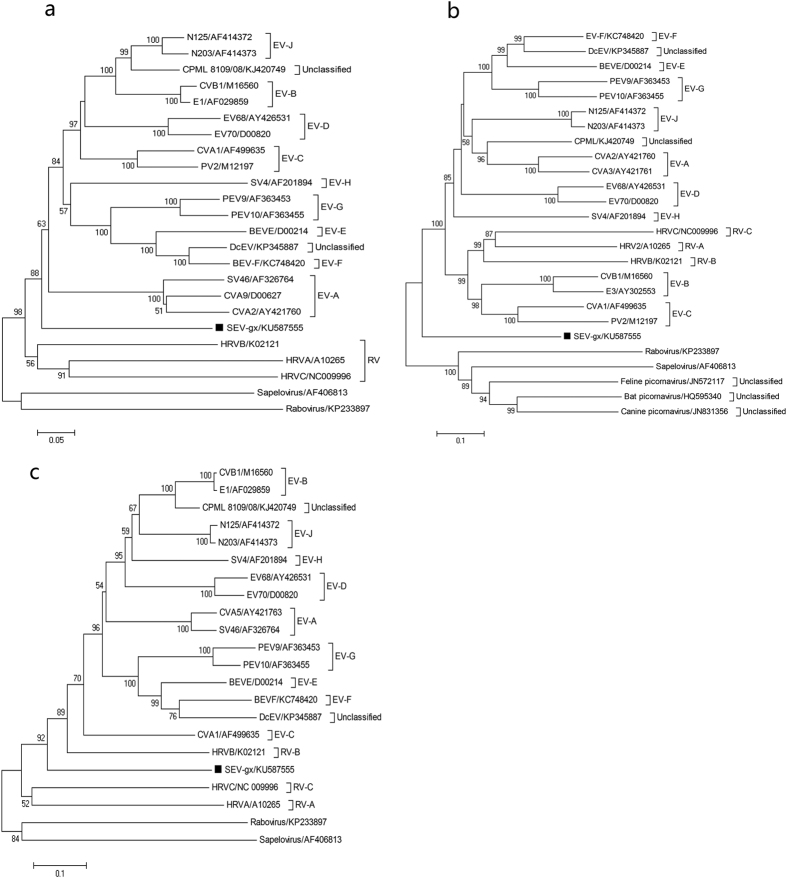
Phylogenetic analyses of SEV-gx are constructed based on the complete amino acid sequences of P1 (**a**), 2C (**b**), 3CD (**c**) with other picornaviruses using the neighbour-joining method with datasets of 1,000 replicates in MEGA 4.0 software. The position of SEV-gx is marked by ◾.

Phylogenetic trees were constructed based on the complete aa sequences of P1, 2C and 3CD of SEV-gx and other representative picornaviruses. In the P1 region, SEV-gx formed a single monophyletic tree related to the cluster of picornaviruses including EV, Rabovirus, Sapelovirus and avian picornaviruses, which was close to the root between the genera EV and Rabovirus, with 100% bootstrap support (Fig. 3a). In the conserved 2C region, SEV-gx formed an independent tree between human rhinovirus species B and C in the EV genus, showing it to be most closely related to human rhinovirus species B (Fig. 3b). However, in the conserved 3CD region, SEV-gx formed a monophyletic tree between human rhinoviruses and EVs, showing it to be relatively closely related to human rhinoviruses, with 89% bootstrap support (Fig. 3c).

## Discussion

EVs are one of the most common viruses infecting humans. Infection of humans and animals by EVs is usually asymptomatic, but some EVs cause severe and occasionally fatal diseases in humans and animals^32^. Interestingly, a growing number of studies have reported that some EVs (EV-A76, EV-D111 and EV-A119) co-circulate in both humans and NHPs^33^,^34^,^35^,^36^ and suggesting that wild NHPs could act as EV reservoirs and sources of future emerging EVs^37^. As such, it is very important to document the presence of divergent EVs among NHPs. However, EVs infecting Chinese NHPs are still rarely reported. Here, we report a novel EV, identified in faecal samples from *Macaca mulatta* at LongHu Mountain in Guangxi Province, China, by Miseq high-throughput sequencing. To the best of our knowledge, this is the first study on the identification of EVs from Chinese wild NHPs.

Genomic characterisation analysis showed that SEV-gx contains a type I IRES and has no L protein. VP0 protein is assumed to be cleaved into VP4 and VP2 at the cleavage site of Lys/Ser. The VP1 protein possesses the PAL(QT)A(AV)ETG and M(FIY)VPPG motifs. 2A protein has the catalytic triad of His-Asp-Cys and the conserved CXCG motif that makes it function as a protease to cleave VP1/2A. 2C protein has the NTPase motif GXXGXGKS and the helicase motif DDLXQ. 3C^pro^ has the catalytic triad of His-Glu-Cys and conserved CXCG motifs, which enables it function as a chymotrypsin-like protease. 3D^pol^ has the RNA-binding domain (KFRDI) and the conserved motifs of RNA-dependent RNA polymerases. All of these findings demonstrate that the structural features of SEV-gx are similar to those of members of the EV genus in the family Picornaviridae.

The Picornaviridae Study Group (PSG) guidelines state that members of a species of the genus should share <70.0% aa identity in the polyprotein, <60.0% aa identity in P1 and <70.0% aa identity in 2C + 3CD, while members of different genera should share less than 40.0%, 40.0% and 50.0% aa in P1, P2 and P3, respectively. From the sequence alignment of SEV-gx, we know that it is most closely related to those of species EV-J (simian EVs) (43.0–44.1% aa identity in P1, 52.3–55.2% aa identity in P2, 61.1–62.7% aa identity in P3 and less than 64.0% aa identity in 2C/3CD combined), which meets the criteria for a different species in the EV genus. Phylogenetic analysis of P1, 2C and 3CD between SEV-gx and other picornaviruses showed that SEV-gx formed a monophyletic tree in the genus EV. Therefore, SEV-gx should be classified as a member of a distinct species (tentatively named EV-K) in the genus EV.

SEV-gx was detected in approximately 10.0% of *Macaca mulatta* stool samples, suggesting that it is common in the local rhesus macaque population. The 100.0% nt identity of all of the SEV-gx VP1 sequences showed that the virus is stably circulating locally. Except for SEV-gx, the viral metagenomic analysis in this study did not show any other EV-related viruses in *M. mulatta*, which contrasts with recent reports about the discovery of widely diverse EVs infecting monkeys in the wild^18^,^35^. From the phylogenetic analysis of the P1 region, SEV-gx was somewhat distinct from other known EVs and close to the *raboviruses*^38^ and *sapeloviruses*, which implied that SEV-gx may be a special evolutionary intermediate between EVs and raboviruses/sapeloviruses. Meanwhile, the relatively large distance of SEV-gx from other EVs also suggested that the diversity of EVs in NHPs would be much broader than previously recognised. However, the phylogenetic analysis of the 2C/3CD region was not consistent with that for P1, and showed that SEV-gx was most closely related to human rhinoviruses, which indicated that SEV-gx and human rhinoviruses may share a common SEV-gx-like ancestor. Since the 2C/3CD region is conserved across all of the picornaviruses, it will most likely reflect the true phylogeny between SEV-gx and other picornaviruses. These findings about the special genome of SEV-gx should facilitate future research about the evolution of EVs.

Previous studies have suggested that the transmission of some EVs from wild NHPs to humans may have occurred recently^33^,^34^,^35^,^36^. In fact, NHPs have been indicated as a virus reservoir and have spread some important viral pathogens to humans, including Ebola/Marburg viruses and human immunodeficiency virus^39^,^40^. Thus, it would be reasonable to expect that wild NHPs could play the role of a reservoir and/or source of future emerging EVs with unpredictable symptomatology in humans. The spread of SEV-gx from local NHPs to adjacent habitats can also not be ruled out. Therefore, it is important to perform further studies, including serological assays, on humans in local areas to predict their risks of infection. Furthermore, more studies on virus species in wild animals are necessary to prevent and control human viral diseases, in view of the major environmental changes currently faced by wild animals and humans.

## Materials and Methods

### Specimens

A total of 280 faecal samples were randomly collected from *Macaca mulatta*at LongHu Mountain in Guangxi Province, China, from January to May, 2014. All samples were transported to our lab on dry ice and stored at −80 °C until further analysis.

### Sample extraction and high-throughput sequencing

Total nucleic acids were extracted from the 280 samples that were diluted with phosphate-buffered saline (PBS) (1:10 w/v ratio) and passed through 0.45-μm and 0.22-μm filters, using a QIAamp Viral Mini Kit (Qiagen, Hilden, Germany), in accordance with the manufacturer’s instructions. Viral nucleic acid libraries were constructed by sequence-independent random RT-PCR amplification. Then, the PCR products were sequenced using an Illumina Miseq 2500 platform (Illumina, San Diego, CA, USA). Initial sequencing data were analysed using the customised informatics pipeline Virus Hunter, as described previously^41^.

### Detection of SEV-gx and amplification of the complete VP1 region

The presence of SEV-gx was confirmed in 280 faecal samples from *Macaca mulatta* by reverse transcription nested PCR (RT-nested PCR) with PrimeScript One Step RT-PCR Kit (Takara, Tokyo, Japan), based on the sequences obtained by Miseq sequencing. Complete VP1 sequences were amplified for the SEV-gx-positive samples, using PrimeScript One Step RT-PCR Kit (Takara). All of the amplifications were achieved under the following conditions: 50 °C for 30 min, and 94 °C for 5 min, followed by 35 cycles (94 °C for 30 s, 53 °C for 30 s and 72 °C for 1 min) and then 72 °C for 7 min. The RT-PCR products were electrophoresed and purified on a 1.5% agarose gel. The sequences were determined using the Big-Dye terminator cycle sequencing kit and the ABI Prism 310 Genetic Analyzer (Applied Biosystems, Foster City, CA, USA). All of the primers used are listed in Table S1.

### Whole-genome sequencing

To obtain the complete genome sequence of SEV-gx, the genome-walking Kit (Takara) was used to amplify the unknown sequences, and the full terminal sequences were determined by repeated amplification and sequencing using the 5′ and 3′ Rapid Amplification of cDNA Amplification Kit (Clontech, Mountain View, CA, USA), in accordance with the manufacturer’s instructions. The specific primers used here were based on the obtained contig and newly amplified sequence. Three long overlapping fragments were amplified to confirm the final genomic sequence using LA-Taq DNA polymerase (Takara). All primers used here are shown in Table S1.

### Sequence analysis

The sequence of SEV-gx was analysed by sequence alignment with other EVs and representative picornavirus sequences, using Clustalx (ver. 1.83). Pairwise nt and aa identities between SEV-gx and other picornaviruses were calculated using DNAMAN software. Cleavage sites of SEV-gx were predicted based on the alignment of EVs and other picornavirus sequences.

### RNA structure prediction of the 5′-UTR

The 5′-UTR RNA secondary structure of SEV-gx was predicted using consecutive fragments of the complete nt sequence of the 5′-UTR and a thermodynamic folding energy minimisation algorithm with RNA structure software (ver. 5.3). The graph was integrated using RnaViz software (ver. 2.0.3).

### Phylogenetic analysis

To determine the phylogenetic relationship of SEV-gx, the aa sequences of the P1 and conserved 2C/3CD regions were aligned between SEV-gx and other EVs and picornavirus strains using Clustalx (ver. 1.83), and MEGA 4.0 software was then used to construct phylogenetic relationships by the neighbour-joining method with datasets of 1,000 replicates.

## Additional Information

**How to cite this article**: Ao, Y.- *et al*. Identification of a Novel Enterovirus Species in Rhesus Macaque in China. *Sci. Rep.* **6**, 28526; doi: 10.1038/srep28526 (2016).

## Supplementary Material

Supplementary Information

## Figures and Tables

**Table 1:** Comparison of nucleotide and amino acid sequences between SEV-gx and other picornaviruses.

Rejion^a^	Identity (%)									Others^b^
	EV-A	EV-B	EV-C	EV-D	RV (A–C)	EV-(E–F)	EV-G	EV-J	EV-H	
5′UTR	50.1–57.1	51.1–56.0	48.5–54.9	50.8–52.2	38.3–55.1	40.1–43.6	49.2	53.8–56.1	57.1	
**P1**	39.3–43.8	40.7–44.3	38.3–41.5	41.5–42.3	32.2–41.4	43.1–42.7	41.5–42.3	43.0–44.1	39.6	≤25.4
VP1	29.3–32.1	29.5–32.3	23.8–31.2	26.7–30.4	16.9–30.3	32.1–32.3	26.5–29.6	28.3–29.4	28.4	<17.0
**P2**	50.5–52.5	50.8–52.8	47.7–50.3	49.8–51.5	39.2–42.2	50.9–53.4	48.6–51.2	52.3–55.2	54.0	≤21.6
2A	40.5–45.1	43.1–48.4	42.5–45.8	42.5–47.7	25.3–27.5	45.1–46.4	44.4–45.8	47.1–52.9	47.1	
2C	52.6–54.4	51.1–52.9	51.3–53.0	49.4–51.5	38.0–53.5	50.1–52.4	48.3–50.5	50.0–52.1	51.4	
**P3**	56.7–58.1	57.1–61.3	59.7–61.1	60.1–60.6	46.6–54.5	54.9–59.2	57.6–59.3	61.1–62.7	55.4	≤42.4
3CD	55.5–60.4	58.7–62.3	59.7–61.6	60.4–61.0	46.9–54.6	55.8–60.3	56.9–59.8	61.4–63.4	54.9	
3′UTR	28.8–37.8	27.9–39.8	40.2–46.0	39.8–42.5	23.8–33.8	33.7–43.3	38.8–39.3	25.9–36.2	33.4	

^a^Nucleotide comparisons for UTRs; amino acids comparisons for coding region.

^b^other picornaviruses.
