# Supplementary material for: Identification of a Novel Enterovirus Species in Rhesus Macaque in China
Source: Sci Rep. 2016 Jun 22;6:28526. doi: 10.1038/srep28526 (PMC4916455; doi:10.1038/srep28526)
Supplement: Supplementary Information [file srep28526-s1.doc]

**Identification of a Novel Enterovirus Species in Rhesus Macaque in China**

Yuan-yun Ao^1^, Jie-mei Yu^1^, Cui-yuan Zhang^1^, Yun-yun Xin^2^, Li-li Li^1^, Zhao-jun Duan^1＊^

**Supporting Information**

**Table S1.** List of the primers used for cDNA synthesis and polymerase chain reaction.

| ID Primers | Sequence 5'-3' | Position | Product length | Note |
| --- | --- | --- | --- | --- |
| EVF1 | tggcagcagacccttgtga | 4781-4798 | 404bp | A1 |
| EVR1 | agctgttgtcagtgtctgg | 5184-5166 |
| EVF2 | agagcaacaggagttagaa | 4870-4888 | 193bp |
| EVR2 | agtcagttgcctggagca | 5062-5045 |
| VP1F | tgactgccccactacagctaag | 2223-2244 | 1111bp | A2 |
| VP1R | tggatcatcttctggatccagga | 3333-3311 |
| 5race | ctccttagtagcgcaatagcgccttcac | 463-436 | 463bp | B2 |
| W1sp1 | acagctgttgtcagtgtctgga | 5186-5165 | 2204bp | B1 |
| W1sp2 | cttctgagtcagttgcctggagca | 5068-5044 |
| W1sp3 | ctcattctaactcctgttgctct | 4892-4870 |
| W2sp1 | acatgctataagcattagccactga | 2929-2905 | 849bp | B1 |
| W2sp2 | gaacacactggggtttgttgcag | 2859-2837 |
| W2sp3 | caggaggcacatacataacctgg | 2794-2772 |
| W3sp1 | gacaaagtcatcacaagcagacac | 2280-2257 | 1659bp | B1 |
| W3sp2 | tggtgattctccctgaagtggagt | 2191-2168 |
| W3sp3 | tgaccaatgtagcagttgactgt | 2098-2076 |
| W4sp1 | tgccgtattggcaatcaataccaca | 5604-5628 | 1338bp | B1 |
| W4sp2 | atgtaacccagtacggagcattga | 5660-5683 |
| W4sp3 | gagagcaggtcagtgtggtggtgt | 5733-5756 |
| 3race | tcaagaggtacttcagagctgatgaagag | 7025-7053 | 372bp | B2 |
| EV1F | acatgggccagcccaccacac | 1-21 | 3140bp | C |
| EV1R | ggagctttgtagggtgcctgtctg | 3140-3117 |
| EV2F | gtgtctgcttgtgatgactttgtc | 2257-2280 | 3041bp | C |
| EV2R | tgagcagttctaagagttggcac | 5297-5275 |
| EV3F | agagcaacaggagttagaatgagt | 4870-4893 | 2414bp | C |
| EV3R | ccgtccaaccactgattacgtag | 7283-7261 |
| Oligo | aagcagtggtatcaacgcagagtacgcggg | 5'end |  | D1 |
| 3 Primer A | aagcagtggtatcaacgcagagtac(t)30vn | 3'end |  | D1 |
| 5 Primer A | (t)25 v n | 3'end |  | D1 |
| UPM | ctaatacgactcactatagggc- aagcagtggtatcaacgcagagt | 3'/5'end |  | D2 |
| NUM | aagcagtggtatcaacgcagagt | 3'/5'end |  | D2 |

Note: A1: Nested PCR primers used for confirmation and detection of SEV-gx;

A2: Primers used for amplification of VP1 of SEV-gx;

B1, B2: Specifical primers used for amplification of the extreme 5' and 3' end of the genome;

C: Primers used for confirmation of the full-length sequence;

D1: Primers used for cDNA synthesis froma SMART RACE cDNA amlification Kit (Clontech);

D2: Primers used for 3'/5'end amplification from a SMART RACE cDNA amplification Kit (Clontech).
